# Treatment of chronic bullous disease of childhood with dupilumab after dapsone-induced methemoglobinemia in a 2-year-old female patient

**DOI:** 10.1016/j.jdcr.2023.08.011

**Published:** 2023-08-21

**Authors:** Faraz Yousefian, Fatma Zeynep Deligonul, Lisa Swanson

**Affiliations:** a Goodman Dermatology, Roswell, Georgia; b University of Incarnate Word School of Osteopathic Medicine, San Antonio, Texas; c Ada West Dermatology, Boise, Idaho

**Keywords:** biologics, chronic bullous disease of childhood (CBDC), dapsone, dupilumab, methemoglobinemia

*To the Editor:* We read with interest the report by Almuhanna et al^1^ on the remarkable response to the off-label use of dupilumab in a 7-year-old boy with linear IgA bullous dermatosis. This case report highlights the challenges of treating linear IgA bullous dermatosis with the current conventional treatment. We aim to expand upon this finding regarding the successful treatment of chronic bullous disease of childhood (CBDC) with dupilumab after dapsone-induced methemoglobinemia in our patient.

A 2-year-old female patient was referred to our clinic with tense bullae in rosette configurations and erosions distributed on the trunk, back, extremities, ears, buttocks, and labia majora. Her body weight was 16 kg, and she was not responsive to oral prednisolone 35 mg daily for the past 4 days (Fig 1). Moreover, she did not have systemic symptoms. Biopsy was considered but not performed because of the classic clinical findings of CBDC. The patient was tapered off prednisolone and simultaneously started on oral dapsone 32 mg daily after confirmation of normal complete blood count, comprehensive metabolic panel, and glucose-6-phosphate dehydrogenase (G6PD) level laboratory results. After 5 days of dapsone treatment, physical examination revealed blue discoloration of the face, lips, and tongue without any improvement of prior lesions (Fig 2).

**Fig 1:**
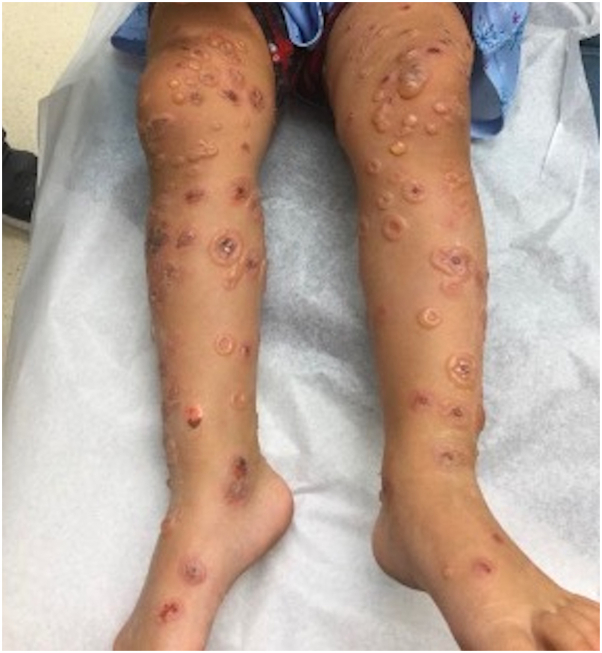
Physical examination revealing tense bullae in rosette configurations and erosions distributed on the lower extremities.

**Fig 2:**
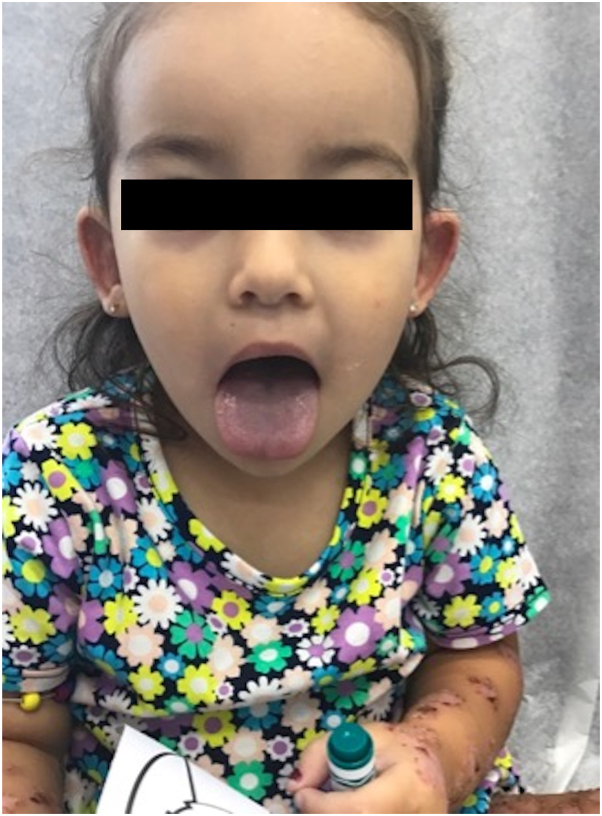
Physical examination revealing blue discoloration of the face, lips, and tongue after 5-day treatment with dapsone.

Consequently, laboratory blood test revealed an elevated methemoglobin level of 20.9% (normal range, 0.0%-1.5%). The patient was diagnosed with dapsone-induced methemoglobinemia and admitted to pediatric intensive care unit for methylene blue treatment. Her parents were reluctant to consider other potential conventional options such as cyclosporine or mycophenolate mofetil because of the trauma of dealing with the methemoglobinemia from the dapsone. The patient was started on dupilumab 300 mg monthly injection and oral prednisolone 45 mg daily. The 8-week follow-up revealed significant improvement in lesions with prednisolone tapering (Fig 3). The patient responded well to stopping prednisolone completely, and she is currently on dupilumab only.

**Fig 3:**
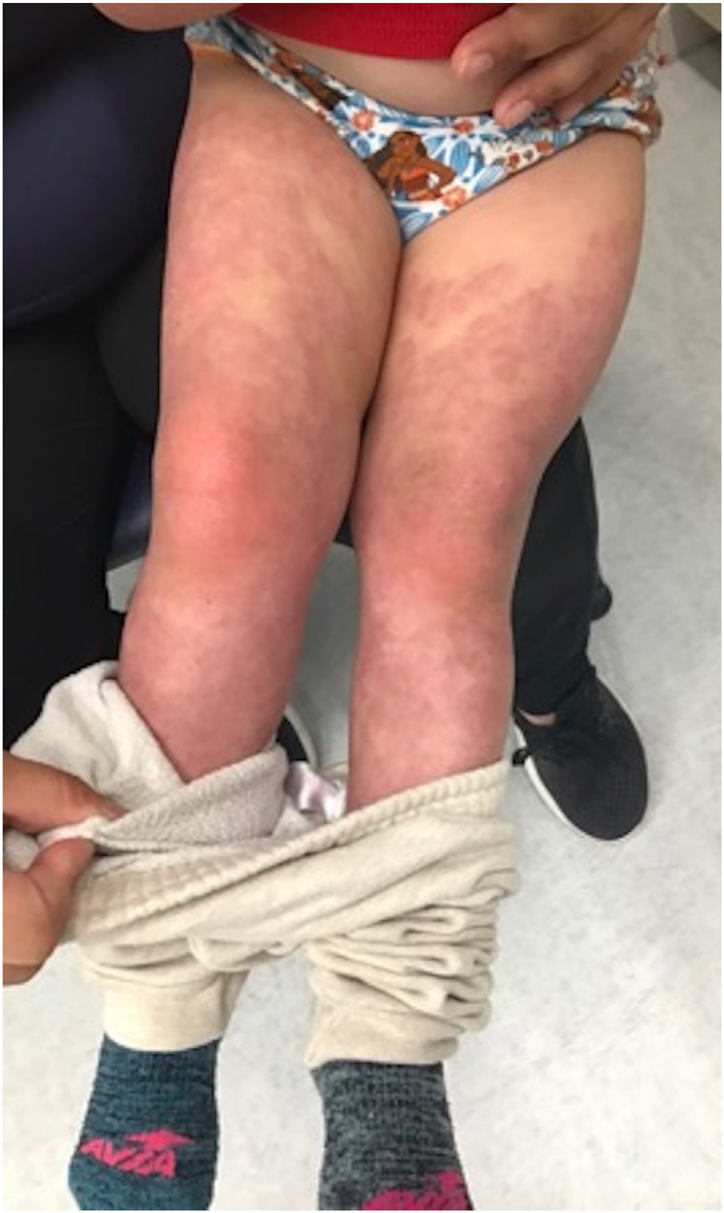
Eight-week dupilumab after treatment.

Although systemic dapsone is commonly considered first-line treatment for CBDC, common and severe adverse effects include hemolytic anemia and methemoglobinemia, with an increased risk in patients with G6PD deficiency.^2^ One study of 138 cases of acquired methemoglobinemia showed that dapsone accounted for 42% of cases.^3^ Methemoglobinemia can manifest as cyanosis, dyspnea, lethargy, and headache; the primary treatment of acquired methemoglobinemia is methylene blue, and ascorbic acid is the treatment of choice where methylene blue is unavailable or contraindicated (eg, in patients with G6PD deficiency).^4^^,^^5^

Dupilumab is not currently Food and Drug Administration-approved for the treatment of CBDC, but due to its relative safety profile, it has been reported to effectively treat many dermatological and bullous conditions off-label,^6^ including a severe case of IgA bullous pemphigoid in an adult patient.^7^ In a multicenter case series with dupilumab as a novel therapy for bullous pemphigoid, disease clearance or satisfactory response improved in 92.3% (12 of 13) of patients within a median of 2 months of treatment initiation, with 5 patients reporting improvement in pruritus and bullae within 1 month.^8^ Our findings show that dupilumab may be an effective treatment alternative for pediatric patients who are at risk of developing methemoglobinemia with systemic dapsone or have already developed it.

## Conflicts of interest

Author Swanson is speaker for Abbvie, Almirall, Amgen, Incyte, Janssen, Lilly, Novartis, Ortho Dermatologics, Pfizer, and Sanofi-Regeneron and consultant for Janssen, Lilly, Ortho Dermatologics, Pfizer, Sanofi-Regeneron, Leo, Novan, Arcutis, and Dermavant. Authors Yousefian and Deligonul have no conflicts of interest to declare.
